# Cumulative effects of natural and anthropogenic disturbances on the forest carbon balance in the oil sands region of Alberta, Canada; a pilot study (1985–2012)

**DOI:** 10.1186/s13021-020-00164-1

**Published:** 2021-01-19

**Authors:** C. H. Shaw, S. Rodrigue, M. F. Voicu, R. Latifovic, D. Pouliot, S. Hayne, M. Fellows, W. A. Kurz

**Affiliations:** 1 Canadian Forest Service, Natural Resources Canada, Edmonton, AB Canada; 2 Canadian Centre for Remote Sensing, Natural Resources Canada, Ottawa, ON Canada; 3 Science and Technology Branch, Environment and Climate Change Canada, Ottawa, ON Canada; 4 Science and Technology Branch, Environment and Climate Change Canada, Gatineau, PQ Canada; 5 Canadian Forest Service, Natural Resources Canada, Victoria, BC Canada

**Keywords:** Environmental impact assessment, Model, Greenhouse gases, Cumulative effects, GCBM

## Abstract

**Background:**

Assessing cumulative effects of anthropogenic and natural disturbances on forest carbon (C) stocks and fluxes, because of their relevance to climate change, is a requirement of environmental impact assessments (EIAs) in Canada. However, tools have not been developed specifically for these purposes, and in particular for the boreal forest of Canada, so current forest C assessments in EIAs take relatively simple approaches. Here, we demonstrate how an existing tool, the Generic Carbon Budget Model (GCBM), developed for national and international forest C reporting, was used for an assessment of the cumulative effects of anthropogenic and natural disturbances to support EIA requirements. We applied the GCBM to approximately 1.3 million ha of upland forest in a pilot study area of the oil sands region of Alberta that has experienced a large number of anthropogenic (forestry, energy sector) and natural (wildfire, insect) disturbances.

**Results:**

Over the 28 years, 25% of the pilot study area was disturbed. Increasing disturbance emissions, combined with declining net primary productivity and reductions in forest area, changed the study area from a net C sink to a net C source. Forest C stocks changed from 332.2 Mt to 327.5 Mt, declining by 4.7 Mt at an average rate of 0.128 tC ha^−1^ yr^−1^. The largest cumulative areas of disturbance were caused by wildfire (139,000 ha), followed by the energy sector (110,000 ha), insects (33,000 ha) and harvesting (31,000 ha) but the largest cumulative disturbance emissions were caused by the energy sector (9.5 Mt C), followed by wildfire (5.5 Mt C), and then harvesting (1.3 Mt C).

**Conclusion:**

An existing forest C model was used successfully to provide a rigorous regional cumulative assessment of anthropogenic and natural disturbances on forest C, which meets requirements of EIAs in Canada. The assessment showed the relative importance of disturbances on C emissions in the pilot study area, but their relative importance is expected to change in other parts of the oil sands region because of its diversity in disturbance types, patterns and intensity. Future assessments should include peatland C stocks and fluxes, which could be addressed by using the Canadian Model for Peatlands.

## Background

Cumulative effects, defined by the Canadian Council of Ministers of the Environment as “a change in the environment caused by multiple interactions among human activities and natural processes that accumulate across space and time” [1], are of broad scientific interest [2–7] and in Canada are at the forefront of scientific and technical investigations in the context of environmental impact assessments (EIAs) [8, 9], policy development [10–12] and monitoring approaches [13, 14]. These considerations apply at both federal and provincial levels in Canada [15]. As of 1995, cumulative effect assessments are required for federal impact assessments [16] and as of 1993 for EIAs in Alberta [17, 18]. These assessments, whether conducted at project or regional scales, require the identification of a suite of important issues for the assessment area and the associated valued ecosystem components (VECs) defined as “components of the environment (biophysical and human) that are identified as important ecologically, socially, or economically and are the focus of attention in environmental assessment” [15]. In the pilot study described here, the issue of interest is climate change and the associated VECs of interest are carbon (C) storage and greenhouse gas (GHG) emissions and removals from the upland forested land-base as affected by anthropogenic (e.g., oil and gas development, forestry) and natural (e.g., wildfire, insect outbreaks) disturbances over time (1985–2012).

In 2003, a Federal-Provincial-Territorial Committee on Climate Change and Environmental Assessment released a guidance document [19] to support the integration of climate change considerations into environmental assessments in Canada [20]. The guidance describes two approaches to incorporating climate change in an environmental assessment; effects of a project on climate change (mainly contributions to GHG emissions), and effects of climate change on the project. Greenhouse gas emissions should be included in both project-level assessments [19] and regional strategic environmental assessments [15] that consider climate change but, because GHGs are transboundary and important to a global environmental issue (i.e., climate change), their importance is greatest for regional strategic assessments.

Under Canada’s Impact Assessment Act (IAA), the assessment of a designated project must consider any cumulative effects that are likely to result from the project, in combination with other physical activities that have been or will be carried out [21]. The IAA also requires that impact assessments of designated projects take into account the extent to which the effects of the project hinder or contribute to the Government of Canada’s ability to meet its commitments to reduce future GHG emissions. The Government of Canada published a strategic assessment of climate change, which outlines GHG and climate change information requirements for projects under the IAA [22].

Canada is regarded as a leader in recognizing climate change considerations in an EIA [23] and guidance documents state that practitioners should seek to describe the project's direct and indirect GHG emissions and related effects, including possible large-scale impacts on C sinks (e.g., impact on forests) [19]. This is highly relevant in the oil sands region (OSR) because it is located in the heart of Canada’s large boreal forest that plays a significant role in the national and global GHG balance [24]. If boreal forest C dynamics are significantly altered by the cumulative effects of anthropogenic and natural disturbances, then these disturbances are a major contributor to the GHG emissions and C storage VECs and should be included in EIAs in the region. Typically, EIAs considering climate change impacts include industrial emissions (e.g., [25, 26]) because they are the largest source of GHG emissions associated with a project, but they typically do not include changes to the forest that also can affect the GHG balance and contribute to cumulative effects. In cases where contributions to GHGs from the forest land-base have been included in an assessment, the approach to estimation has been simplified, and has not included changes over time or accounting for years in which the forest land remains un-forested, and therefore does not take up C from the atmosphere (e.g., [27]). Although tools have not been specifically developed for EIAs to include cumulative effects on forest C (and therefore GHG emissions and removals, and C stocks) here we demonstrate how a new version of the Carbon Budget Model of the Canadian Forest Sector (CBM-CFS3) [28], an existing forest C modeling framework, can be used to quantify the cumulative effects of anthropogenic and natural disturbances on the forest land-base and their effects on GHG emissions, the net C balance, and C storage.

The CBM-CFS3 [28, 29] is a recommended resource for EIA practitioners [19] and is the core model used in Canada’s National Forest C Monitoring Accounting and Reporting System [30, 31]. The CBM-CFS3 is consistent with the Intergovernmental Panel on Climate Change (IPCC) guidelines for estimating GHG emissions and removals from land use, land-use change and forestry (see Kurz et al. [28]). It is an annual time-step, stand-level, upland forest (all productive forest excluding those located in wetlands or peatlands) C model that is driven by forest inventory and merchantable yield curves commonly available in the forest sector. Non-biomass pools (e.g., snags, downed wood, litter and soil) are modelled using turnover and decomposition functions, and emissions estimation is based on C stock changes. The CBM-CFS3 simulates natural (e.g., wildfire, insect damage) and anthropogenic (e.g., forest harvesting, land-use change) disturbance effects by using a disturbance matrix (DM) [32, 33] that defines the proportion of C in each ecosystem pool (represented in the model) that is transferred to other pool(s) (e.g., live biomass to a dead organic matter pool), to the atmosphere, or to harvested wood products as a result of a disturbance, in the year that the disturbance occurs. Disturbance matrices are well developed for stand replacing wildfires, insect disturbance events and harvesting, but are generalized for oil and gas activities [29, 30]. Therefore, DMs for the common oil and gas exploration and development disturbance types for upland forests were developed as part of this pilot study.

The availability of large volumes of remotely-sensed data and advances in computer science have enabled the development of a spatially-explicit and scalable version of the CBM-CFS modelling framework, which has been applied at various pixel resolutions ranging from 30 × 30 m on areas of a few million ha in size to 100 × 100 m over areas exceeding 60 million ha [34]. By facilitating the integration of data from multiple sources, a spatially-explicit approach increases transparency, accuracy, consistency, completeness and comparability of estimates [35, 36]. The new Generic Carbon Budget Model (GCBM) builds on the science of the CBM-CFS3 but uses a new computing approach that enables the simulation of C dynamics of landscapes comprising millions of pixels. The CBM-CFS3 used a spatially-referenced approach, where the forest inventory data were associated with polygons of varying size, representing timber and land management regions, with no specific knowledge of where the forest stands or disturbances were located within the region. The spatially-explicit framework of the GCBM allows for grid-based modelling at a scale determined by the user. A spatially-explicit modelling prototype leading to the development of the GCBM has been applied at the scale of photo plots to assess C dynamics on agricultural lands reverting to woody land in Ontario [37], the scale of a watershed to assess the effect of reservoir expansion in British Columbia [38] and the scale of a region to test the integration of spatially-explicit Landsat derived data layers into C accounting with the CBM-CFS3 [39]. The GCBM is suitable for modelling fine-scale oil and gas disturbances in conjunction with coarse-scale disturbance effects from forest harvesting and wildfire over a large landscape area.

The main objective of this pilot study is to demonstrate the value of using an existing tool (GCBM) in providing outputs useful for EIAs by (1) developing and implementing methods that integrate spatially-explicit data on natural and anthropogenic disturbances derived from remote sensing time series and other data sources within a spatially-explicit modelling framework (GCBM), (2) developing DMs for the common oil and gas exploration and development disturbance types for upland forests to more accurately describe the variation in the impacts of different activities on existing C stocks in biomass, dead organic matter and soil C pools, and (3) estimating the cumulative effects of multiple types of oil and gas disturbances, as well as disturbances from harvesting, wildfire, and insects on the C balance, GHG emissions and removals, and C storage in the study area within the OSR.

## Methods

### Study area

**Fig. 1:**
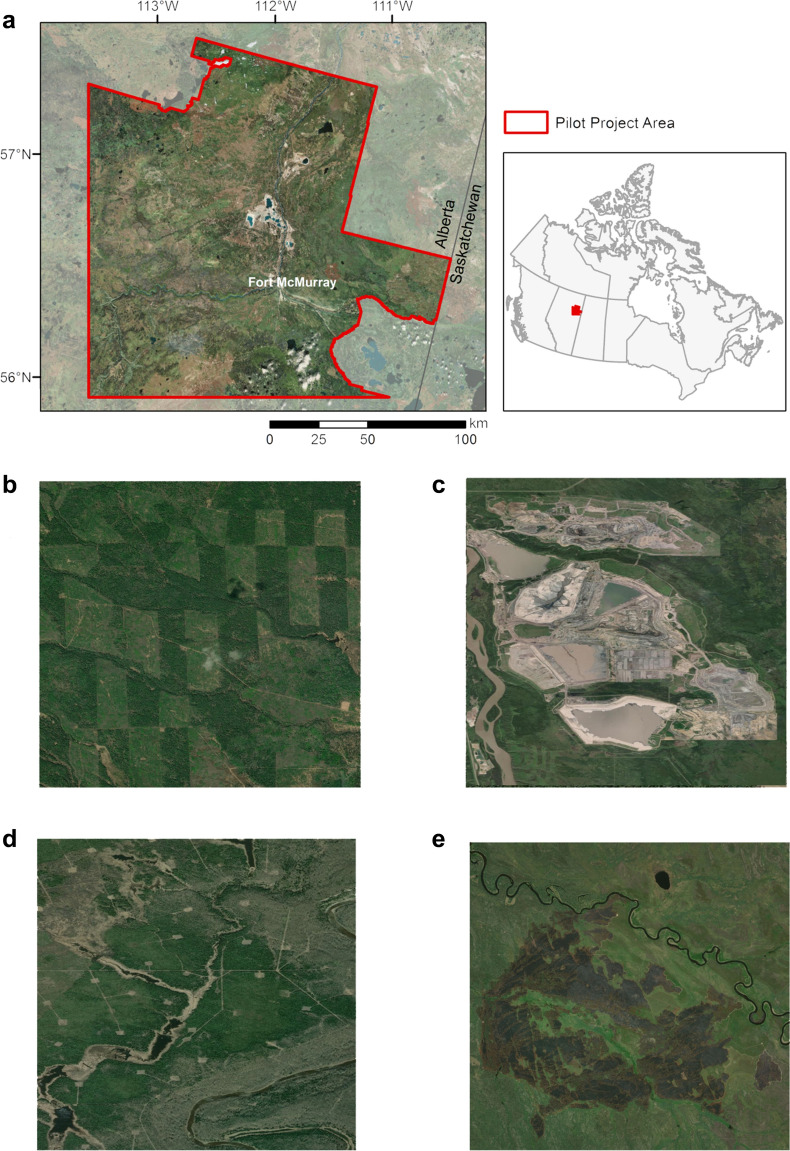
Location of the pilot study area and example disturbance types. **a** The pilot study area (approximately 2.5 Mha) is located in northern Alberta, Canada and includes approximately 1.3 Mha of upland forest, 0.8 Mha of wetlands and 0.4 M ha of other land types (e.g., bare surface, urban development, water). Examples of disturbance types in the region include **b** forest harvesting, **c** surface mining, **d** in-situ oil and gas extraction and **e** wildfire. This study was applied to the upland forest area only

The pilot study area is located in the boreal forest of northern Alberta in the vicinity of the city of Ft. McMurray (Fig. 1). The region is subject to a wide range of natural and anthropogenic disturbances, including intensive oil and gas exploration and development activities, forest harvesting, wildfire and insect outbreaks (Fig. 1, [40–45]). The pilot study area covers 2,482,770 ha where 1,267,725 ha are uplands (the focus of this study) with Luvisolic, Brunisolic and Gleysolic soils and 787,361 ha are wetlands with Organic peatland soils [46, 47]. It is in the Central Mixedwood subregion of the Boreal Mixedwood forest of northern Alberta that has a subhumid to subarid (annual precipitation of 389 mm), cool continental climate (mean annual temperature 1.5° C), with long cold winters and warm summers [46]. In the Central Mixedwood subregion the dominant tree species is trembling aspen (*Populus tremuloides* Michx.) and co-dominant species are balsam poplar (*Populus balsamifera* L.), black spruce (*Picea mariana* (Mill.) BSP), white spruce (*Picea glauca* (Moench) Voss) and jack pine (*Pinus banksiana* Lamb.) [46].

The location of the pilot study area was chosen to represent the suite of disturbance types in the OSR, where the best data layers were available for annual change in disturbances over time, fine-scale disturbances from oil and gas exploration and development, and forest inventory with associated yield curves. The data layers were used to generate spatially-explicit inputs for the GCBM.

### Modelling with the GCBM

**Fig. 2:**
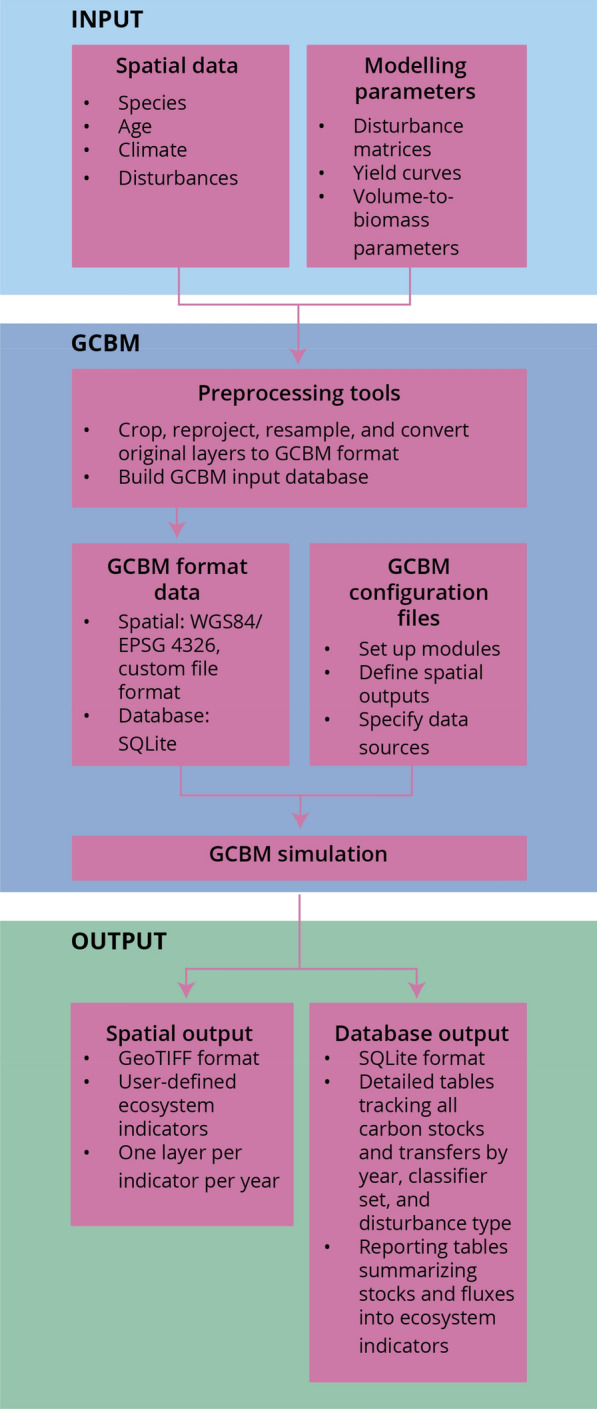
Schematic of processing spatially-explicit simulations with the Generic Carbon Budget Model (GCBM). The carbon science in the GCBM is identical to the Carbon Budget Model of the Canadian Forest Sector (CBM-CFS3) (see Kurz et al. [28])

Modelling of upland forest types was conducted using the GCBM, the next-generation, spatially-explicit version of the CBM-CFS3. The GCBM is composed of science modules linked to the Full Lands Integration Tool (FLINT) framework, an open source, modular, spatially-explicit modelling framework developed jointly by experts from Australia (Mullion Group), Canada (Canadian Forest Service) and the moja global organization [48] (Fig. 2). The science modules in the version of the GCBM used here replicate the representation of C science of the CBM-CFS3 but are linked to the FLINT, which assists in the processing of the spatial information. The CBM-CFS3 simulates the entire landscape one year at a time. In contrast, the GCBM simulates each pixel over the entire time series before moving on to the next pixel, which enables the use of parallel processing computing architecture and allows the spatially-explicit application of the GCBM to much larger landscapes. However, this approach requires that disturbance (and forest management) information is provided as input in spatial layers that define both the year and type of disturbances.

The GCBM uses, as inputs, a combination of a spatial forest inventory (including information like the location of forest types, tree species, stand age), mean annual temperature, and annual disturbance layers, along with non-spatial parameters or parameters that are spatially-referenced to terrestrial ecozones or administrative regions [29]. Model parameters include yield curves, volume-to-biomass conversion coefficients, and disturbance matrices to estimate the annual C balance of a study area. Spatial data for the GCBM are stored in a custom format using the EPSG 4326 / WGS 84 projection [49]. A Python tool is used to crop, re-project, and re-sample spatial layers from their original raster or vector format into the format and projection used by the model. Simulations in this project were conducted at 30 m resolutions. Non-spatial data are stored in an SQLite database populated by a tool that loads a user-provided set of yield curves and a file defining any transitions of stand characteristics following disturbance, and imports other default model parameters from the CBM-CFS3 input databases. In this pilot study the only transitions following disturbance that were represented were reductions in stand growth following seismic line disturbances. Growth for the pixel after disturbance was reduced by the same proportions used for disturbed area for the old (0.27, i.e., established 1960 to 1999) and modern (0.17) seismic lines (see “Results” section “Energy sector disturbances” for rationale).

### Disturbance matrices

The effect of different disturbance types on forest C in the year of the disturbance are modelled in the GCBM (and CBM-CFS3) using disturbance matrices (DMs) [28]. The DMs define the proportions of C that are transferred between model pools, between pools and the atmosphere, and between pools and harvested wood products, at the time of the disturbance. Default DMs used in the GCBM are well developed for wildfires, harvesting, and insect outbreaks [29, 30]. Currently there are two default DMs in the GCBM that could be used to represent disturbances from oil and gas exploration and development. They are intended for application to situations that invoke a land-use change that typically would be associated with large open-pit mining operations used for extraction of resources such as gold, copper, aluminum and coal. The default DMs could have been applied to surface mining in this pilot study, but new DMs were developed in this project for all oil and gas disturbance types, including surface mining, to take advantage of the most recent understanding of the impacts of these disturbance types specific to the OSR.

A team of scientists with expertise in reclamation field research in the OSR and those with expertise in forest C accounting and reporting, and boreal forest C modelling, participated in a 2-day workshop to define the types of DMs required for modelling the effects of oil and gas exploration and development on forest C in the OSR of Alberta. Scientists who attended are leaders in the fields of surface mining reclamation, reclamation land disturbed by in-situ development and exploration disturbances. Others were experts in modeling using the CBM and GCBM and its appropriate implementation for reporting purposes. Initially, discussions were held to identify the suite of oil and gas related disturbance types in the region and factors that would affect fate of woody materials (i.e., proximity to a mill). These disturbance types were distilled into a list for which expert opinion was that sufficient experience and understanding existed to estimate the proportions for C transfers in a DM. These new DMs were then populated using consensus-based values quantifying the proportion of C transfers. Each expert independently estimated the proportion for each transfer in each DM. Values were accepted where the same proportion was estimated by all experts. A discussion was held for each proportion where experts’ initial estimates differed, in order to arrive at a consensus. The intent was to create a suite of DMs for disturbances types that can be identified using the available spatial data layers, and for which there is sufficient understanding of C transfers in response to the disturbances. The DMs were developed for upland situations only, and took into consideration factors such as ecosite type, distance from a mill, permanence, and vintage of seismic lines (see “Results” section “Energy sector disturbances” for details).

### Model inputs

#### Ecological parameters for annual processes

**Fig. 3:**
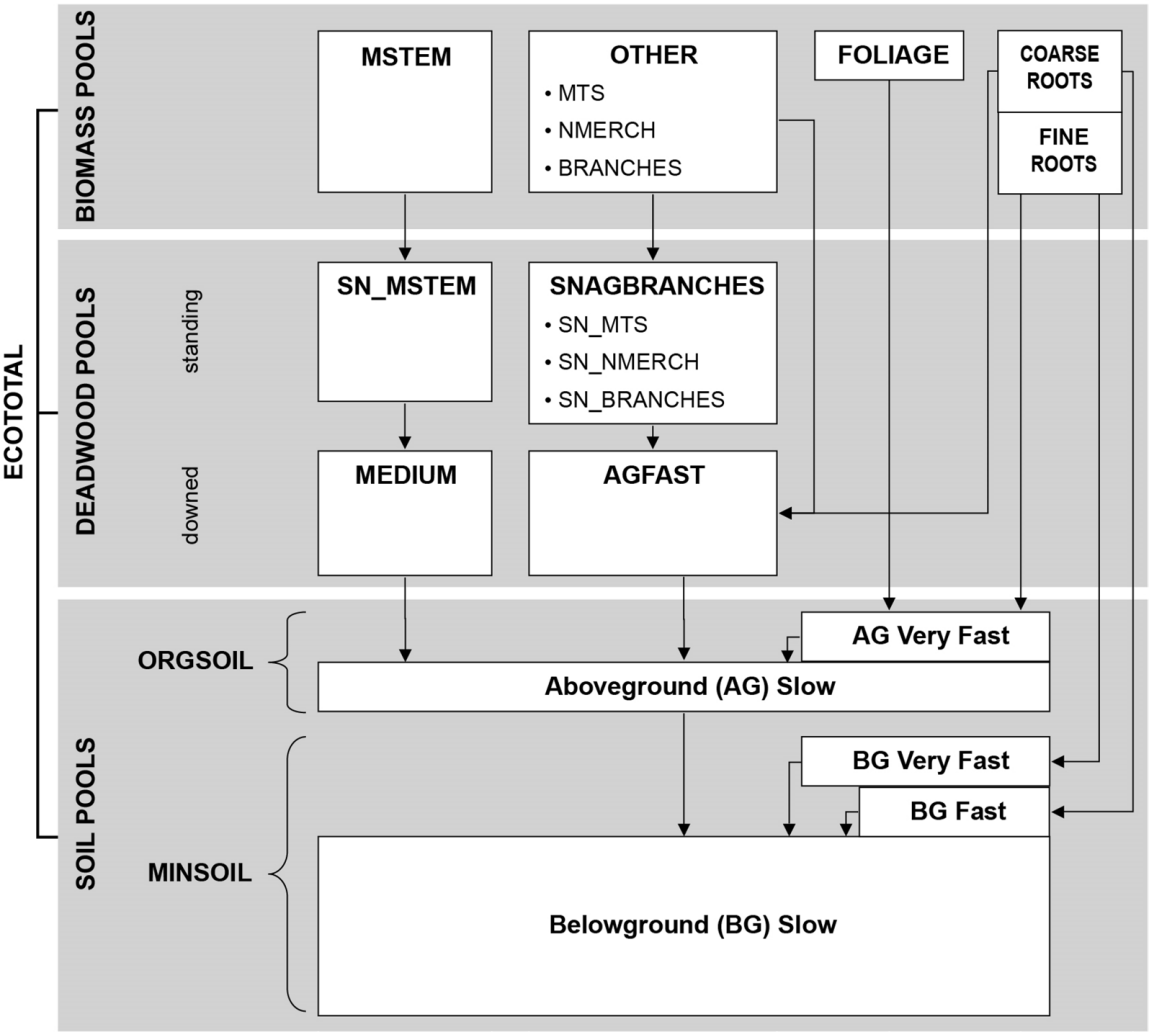
Pool structure of the Carbon Budget Model of the Canadian Forest Sector (CBM-CFS3) which is also used in the Generic Carbon Budget Model (GCBM). Adapted from Shaw et al. [50]). *BIOMASS POOLS: MSTEM* stem bark and wood of merchantable bole for live merchantable trees, *MTS* stem bark and wood in top and stump portion for live merchantable trees, *NMERCH* stem bark and wood in live nonmerchantable trees and saplings; BRANCHES, branch biomass of all live trees (bark and wood), *FOLIAGE* foliage biomass of all live trees, *DEADWOOD POOLS: SN_MSTEM* stem bark and wood of merchantable bole for dead merchantable trees, *SN_MTS* stem bark and wood in top and stump portion for dead merchantable trees, *SN_NMERCH* stem bark and wood in dead nonmerchantable trees and saplings, *SN_BRANCHES* branch biomass of all dead trees (bark and wood), *AGFAST* fine and small woody debris, *MEDIUM* coarse woody debris, *SOIL POOLS* ORGSOIL, LFH and O soil horizons as defined in The Canadian System of Soil Classification [47], *MINSOIL* Organic carbon in mineral soil horizons. Note that consistent with IPCC Guidelines [33] coarse roots (i.e., BGFast C) are reported in the deadwood pool

Annual processes (e.g., decomposition, mortality) determine transfers of C from living biomass to standing and downed deadwood pools, from biomass and deadwood to organic and mineral soil pools, and to the atmosphere are explicitly simulated in the GCBM in the same way as in the CBM-CFS3 (Fig. 3, [28]). Carbon is physically transferred from live to dead pools because of annual biomass turnover and stand mortality (i.e., a yield curve with declining volume). Disturbances transfer C from biomass to dead organic matter (DOM) pools, to the atmosphere, and to harvested wood products. Carbon is also transferred from the slowest decay pool in the organic soil horizons to the slowest decaying pool in the mineral soil, representing a transfer as dissolved organic C. Carbon is moved between DOM pools and to the atmosphere as a result of decomposition. Base decay rates in the GCBM are modified in response to mean annual temperature using a Q_10_ relationship. Base decay rates and Q_10_ vary by DOM pool. Parameters used in this study are those specific to the Boreal Plains ecozone [51] and are specified in Kull et al. [29]. In this study we assume that all C transferred to the forest product sector is instantly oxidized and released to the atmosphere, and thus overestimate the direct emissions from harvest by not estimating C retention in harvested wood products and landfills [30, 31, 34].

#### Inventory data layers

This project integrated an Alberta Vegetation Inventory (AVI, [52]) forest inventory provided by Alberta Pacific Forest Industries, Inc. (AlPac) with two strata defining the softwood and hardwood component. The stratum raster was created by rasterizing the AVI to 30 m spatial resolution and filling the null pixels with species from a basemap derived by the Canadian Centre for Remote Sensing (CCRS) land cover time-series [44]. The basemap was created using the values of the pixels from the time-series that had a forest cover for the first three years of the time-series. The conifer, deciduous and mixedwood forest classes were then converted to the stratum type that corresponded to the dominant AVI stratum intersecting the forest class. To match the yield curve stratification, conifers were labelled PjO-CFM (open and closed jack pine led conifer stands located on fair and medium productivity sites), mixedwoods labelled as AwS_N [aspen (*Populus *spp.) and spruce (*Picea *spp.) mixedwoods stands located on the northern portion of the AlPac's forest management agreement area], and deciduous labelled Aw_comp (pure aspen stand).

Using AlPac’s AVI attributes, every mapped polygon in the AlPac landbase within the pilot study area was post-stratified into one of the 22 yield strata that separate stands or stand groups with different growth characteristics. The key characteristics used to stratify the landbase included species composition, crown closure, timber productivity rating, understory occurrence, and geographical location [53]. The yield strata assignment was computed using a SAS script incorporating rules used to define yield strata (rules taken from Table 10 in [53]).

#### Yield curves

The yield strata were used to link pilot study inventory with yield curves. Yield strata assignments involved the determination of stand characteristics such as overstory and understory broad cover types or leading conifer species [53]. AlPac’s detailed Forest Management Plan is supported by a timber supply analysis, which entails development of yield curves for the various AVI strata. For each yield stratum, defined by AVI stand attributes including species composition, crown closure, site productivity and location (Tables 10 and 12 in [53]), a series of empirical yield curves was constructed based on AlPac’s temporary sample plot and permanent sample plot data. Merchantable yield curves were fit using non-linear regression, applied separately to the total, coniferous, and deciduous, volume-age pairs [53]. Coniferous and deciduous curves generated separately in each yield stratum were matched to species in GCBM. Merchantable yield estimates were projected using 5-year intervals, which were converted to annual estimates using linear interpolation.

#### Disturbance data layers

A merged disturbances time series (1985–2012) was created from the CCRS disturbance time series [44] and the 2012 Alberta Biodiversity Monitoring Institute (ABMI) human footprint [54]. Year and type of disturbance for wildfire, insect disturbances, harvest, and surface mines were taken directly from the CCRS time series. For other disturbance types, the ABMI time series was used to assign disturbance type, and the CCRS time series was used to assign a year to that disturbance type because, at the time this work was completed, ABMI did not provide disturbance year attributes. The CCRS identified approximately 1602 ha of generic disturbances with/without regeneration that included the year of disturbance. These were generally related to oil and gas activities. To assign a year to the ABMI disturbance events, for each ABMI disturbance object, we extracted the mode from the year of first disturbance of the object in the CCRS time series. For well pads, pipelines, roads, residential and industrial sites in ABMI, only the disturbances intersecting with at least one CCRS pixel were included in the merged disturbance time series. For seismic lines, we started by adding the disturbance events that intersected at least one CCRS pixel. We then used the disturbance year of the nearest neighbor to add year attributions to the remaining seismic lines that did not intersect with CCRS pixels. All the CCRS generic disturbances pixels that did not intersect with ABMI disturbances were retained. Disturbances prior to 1985 were excluded from the time series, so effects from surface mining prior to 1985 were not quantified. Seismic line disturbances are narrower than the width of a pixel (approximately 30 m) and were represented by reducing the total disturbance effect of a seismic line type proportional to the disturbed area of the pixel (see “Results” section “Energy sector disturbances”).

## Results

### Energy sector disturbances

**Fig. 4:**
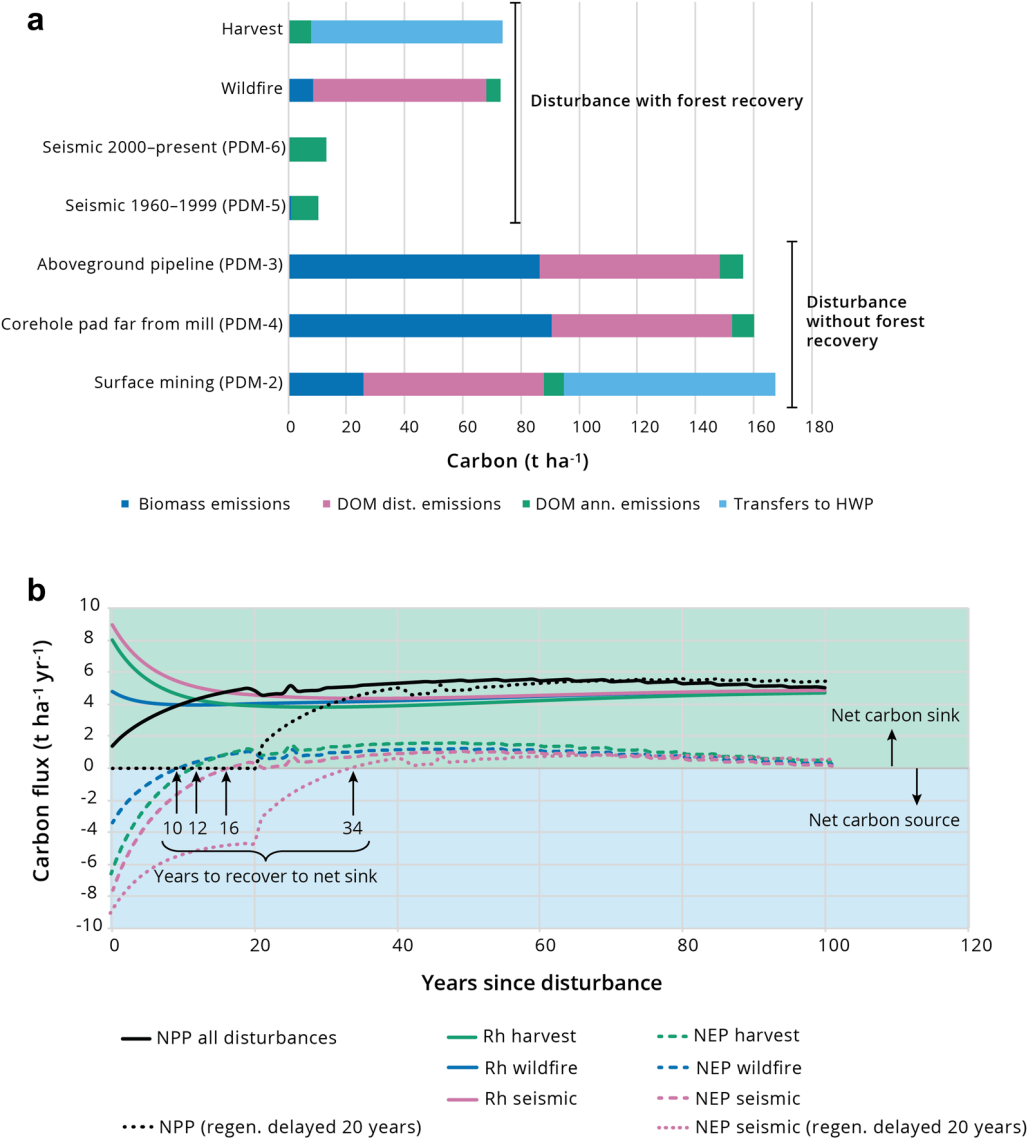
**a** A comparison of carbon (C) transfers out of the ecosystem, either through emissions (combustion or decay) or transfers to harvested wood products (HWP) in the year of disturbance, for different disturbance matrices. The main pathway for removal of C in the year of harvest is transfer to HWP and for wildfire it is combustion of dead organic matter (DOM). Removals of C from the ecosystem in the year initiating surface mining are almost double that of harvest or wildfire because merchantable wood is removed to HWP and the remaining biomass and DOM are piled and burned to clear the land. The amount of C removed in the year of disturbance from aboveground pipelines and corehole pads far from a mill are similar to surface mining but all are emissions to the atmosphere, with no transfer of C to HWP. Emissions of C from seismic lines (regardless of vintage) are similar to one another (low and from enhanced decomposition) and much lower than for the other disturbance types shown here. Results were obtained from application of the disturbance matrices to a theoretical 1 ha of forest lands. PDM, primary disturbance matrix (see Table 1). “DOM dist. Emission” are emissions directly attributed to the disturbance, whereas “DOM ann. Emission” are emissions from the annual process of decay. **b** The relationship between the ecosystem indicators net primary productivity (NPP), heterotrophic respiration (Rh); and net ecosystem productivity (NEP) in the years after disturbance from wildfire, harvest, and old seismic lines (established between 1960–1999) simulated by the Generic Carbon Budget Model (GCBM). When NPP recovers immediately after disturbance, the time to recover to a net sink is influenced by the type of disturbance because it determines the amounts and types of biomass that transfers to DOM pools that subsequently release C as they decay (Rh). Recovery is fastest after wildfire (10 years), followed by harvest (12 years) and then seismic lines (16 years). If recovery of NPP is delayed (e.g., difficulties in initiating reclamation success on seismic lines) for 20 years (in this example) the effect is to further delay recovery of the system to a net sink (34 years vs. 16 years) and total net emissions over the simulation period are higher when reclamation success is delayed

Seven primary DMs (PDMs) were developed to represent 13 energy sector disturbance types in the OSR (Table 1; Additional file 1: Appendix S1). Four of the PDMs (2, 3, 4, 6) were used more than once to represent different disturbance types (DM-types) (e.g., PDM-2 represents DM-Types-2, -5 and -11; Table 1) because knowledge about the effect of these multiple disturbance types on C transfers was insufficient to distinguish their effects. However, the DM types were retained because they can be identified spatially in the disturbance data layers. Disturbance effects were distinguished primarily on the basis of fate of stemwood (piled and burned, left on-site to decompose, or removed for harvested wood products), fate of non-commercial wood including roots (piled and burned or left on-site to decompose), size of disturbance (sub-pixel or not), and degree of soil and forest floor disruption. For example, stemwood from small disturbances far from a mill is more likely to be piled and burned (e.g., DM-Type-4; Table 1; Fig. 4a) compared with large disturbances close to a mill (e.g., DM-Type-5; Table 1), where stemwood is more likely to be harvested and converted to wood products. Disturbances that cause major disruption to the soil (e.g., core hole pads, well pads and permanent roads, pipelines, surface mines) will result in a significant flux of C from the soil to atmosphere in the year of disturbance compared with those that minimally disturb soil (e.g., seismic lines, DM-Type-9; Table 1; Fig. 4). In two cases the disturbance types were unspecified (Table 1) small-scale (i.e., smaller than a LandSat pixel [30 m × 30 m]) non-linear disturbances detected in the CCRS disturbance time series but absent from the ABMI footprint product. DM-Type-10 (PDM-6) developed for “seismic lines 2000 to present”, was used to represent the “unspecified with regeneration” disturbance type (DM-Type-12) under the assumption that these disturbances are likely minimal disturbed areas associated with in-situ development, or if not, the land was disturbed in a similar manner. DM-Type-13 used for “unspecified without regeneration” was also based on PDM-6 but with a slightly greater disturbance effect (see Additional file 1: Appendix S1). We refer to these areas as unspecified in-situ.

**Table 1:** Name and description of disturbance matrices (DMs) developed specifically for modelling oil and gas exploration and development disturbance effects using the Generic Carbon Budget Model (see Additional file 1: Appendix S1 for matrices)

DM type	Primary DM number	Disturbance matrix name	Disturbance matrix description
1	1	OSE Corehole Pads close to mill—not lbh^1^	OSE corehole pads close to the mill not located in the lbh subregion; flat topography; minimal disturbance
2	2	OSE Corehole Pads close to mill–lbh	OSE corehole pads close to the mill located in the lbh subregion; cut and fill used for pad construction
3	3	OSE Corehole Pads far from mill—not lbh	OSE corehole pads far from the mill not located in the lbh subregion; flat topo-graphy; minimal disturbance
4	4	OSE Corehole Pads far from mill—lbh	OSE corehole pads far from the mill located in the lbh subregion; cut and fill used for pad construction
5	2	Permanent Well Pads and Roads close to mill	Permanent well pads, roads or where a large area has been cleared close to the mill
6	4	Permanent Well Pads and Roads far from mill	Permanent well pads, roads or where a large area has been cleared far from the mill
7	3	Pipelines aboveground	Pipelines aboveground
8	4	Pipelines belowground	Pipelines belowground
9	5	Seismic Lines 1960–1999	Seismic lines (1960–1999; no burning; bulldozing, minimal soil disturbance; winter operation; roll-back in same year); 0.27 pixel disturbed
10	6	Seismic Lines 2000 to present	Seismic Lines (2000 to present; no burning; bull-dozing, minimal soil disturbance; winter operation; roll-back in same year); 0.17 pixel disturbed
11	2	Surface Mining	Surface Mining
12	6	Unspecified with regeneration	Unspecified light disturbance where forest regen-eration is apparent in imagery; 0.17 pixel disturbed
13	7	Unspecified without regeneration	Unspecified disturbance where forest regeneration is not apparent in imagery; 0.27 pixel disturbed

See Beckingham and Archibald [44] for description of ecosites in the oil sands region

*OSE* oil sands exploration, *lbh* lower boreal highlands. See section on “Energy sector disturbances” for rationale to use 0.17 and 0.27 multipliers

DMs for seismic lines were customized for modelling with a 30 m × 30 m grid. Because the width of seismic lines are narrower than 30 m they do not disturb the entire pixel. Older seismic lines (for this study 1960–1999) are generally wider than modern seismic lines (for this study 2000 to present). At the outset of this study we assumed a representative value for the width of old seismic lines was 8 m and for modern seismic lines 5 m, based on consultation with regional experts. These assumptions are supported in a review by Dabros et al. [41] who reported that seismic lines from the 1960’s to mid-1990’s were approximately 10 m wide, while modern seismic lines are about 5 m wide. If we assume the seismic line runs the width of a pixel (30 m) this translates into a disturbance of 0.27 and 0.17 of a pixel for old and modern seismic lines, respectively. These multipliers were used to reduce the full effects of the DM designed by the expert working group, to properly reflect the magnitude of the disturbance effect at the scale of a pixel. The same multipliers were used to correctly estimate the area of a pixel affected by the seismic line disturbances, and for reduction in stand growth following disturbance.

Energy sector disturbances with the potential to make the greatest contribution to emissions in the year of the disturbance, tend to be those including combustion of wood and DOM (e.g., PDM-3, -4, and wildfire; Fig. 4a) or that cause major physical disruption of organic and mineral soil organic matter (e.g., PDM-2, -3; Fig. 4a). Disturbances that contribute to emissions over time (decades) are those that cause trees to die, transfer to the forest floor and contribute to emissions through their decomposition over time (Fig. 4b). Disturbances that favour forest stand retention or recovery and maintain or restore forest growth (net primary productivity, NPP; PDM-5, -6; Fig. 4) are associated with lower net emissions (net biome productivity, NBP; Fig. 4b) over time and those that retain the land in an un-vegetated state (DM-Types -3, -7, -11; Table 1) with no restoration of NPP tend to be associated with higher net emissions over time. In this pilot study, we have made the assumption that forest stands begin to recover after disturbance from harvest, wildfire and seismic lines (old and modern) because of insufficient data to determine any lag in the rate of recovery of forests, although this is the subject of research by others [55–57]. Under this assumption, the time to recover to a net C sink (positive values in Fig. 4b) in the first 20 years after disturbance is dependent on disturbance type, and the trajectory of forest stand recovery expressed by the shape and maximum productivity of the yield curve. The lag time to recovery could increase if the disturbance results in conditions that prevent or delay re-establishment of a productive forest. The likely effect of not including these potential additional lag effects in this pilot study is that net emissions (NBP) are slightly underestimated.

### Areas by disturbance type

**Fig. 5:**
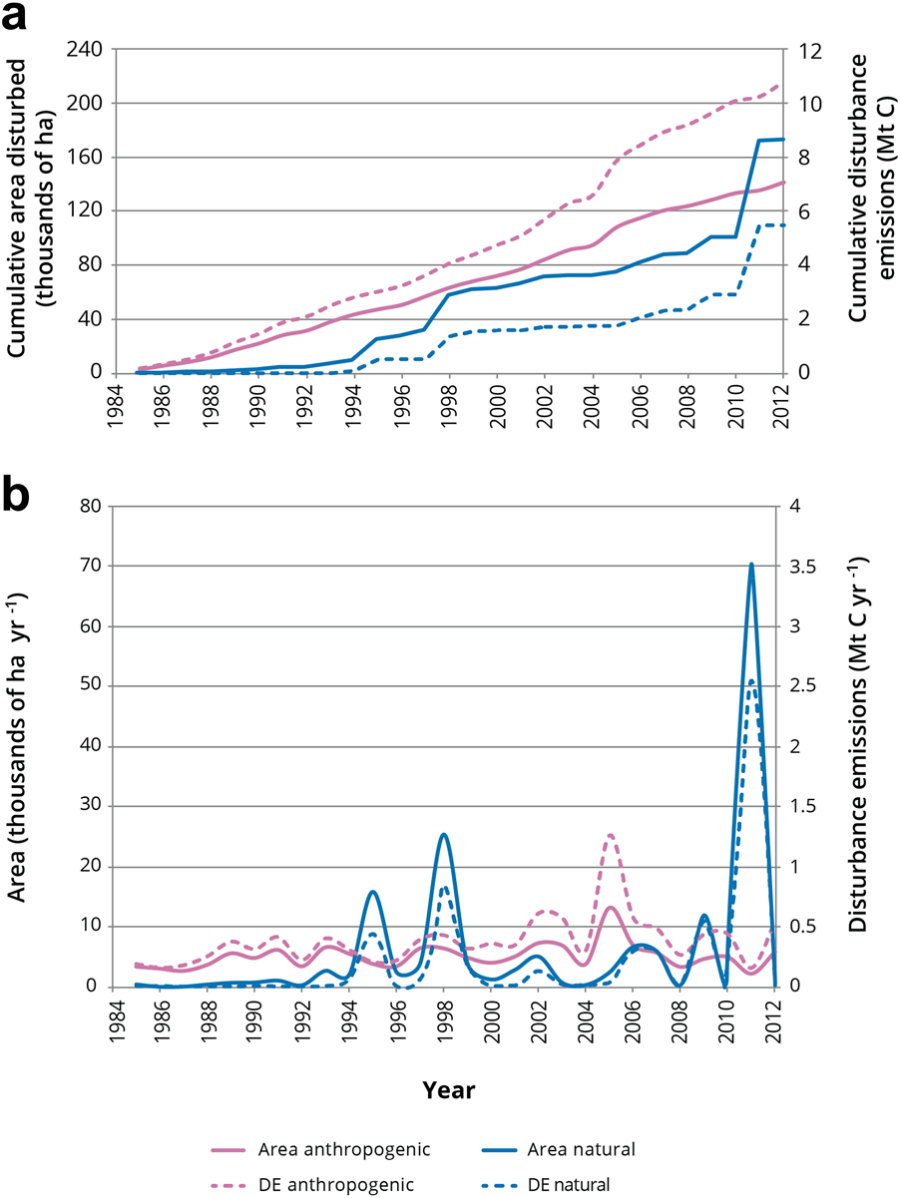
Cumulative disturbance emissions (DE) and area disturbed (**a**), and annual disturbance emissions and area disturbed (**b**) in the pilot study area (1985–2012) from natural and anthropogenic disturbances

**Fig. 6:**
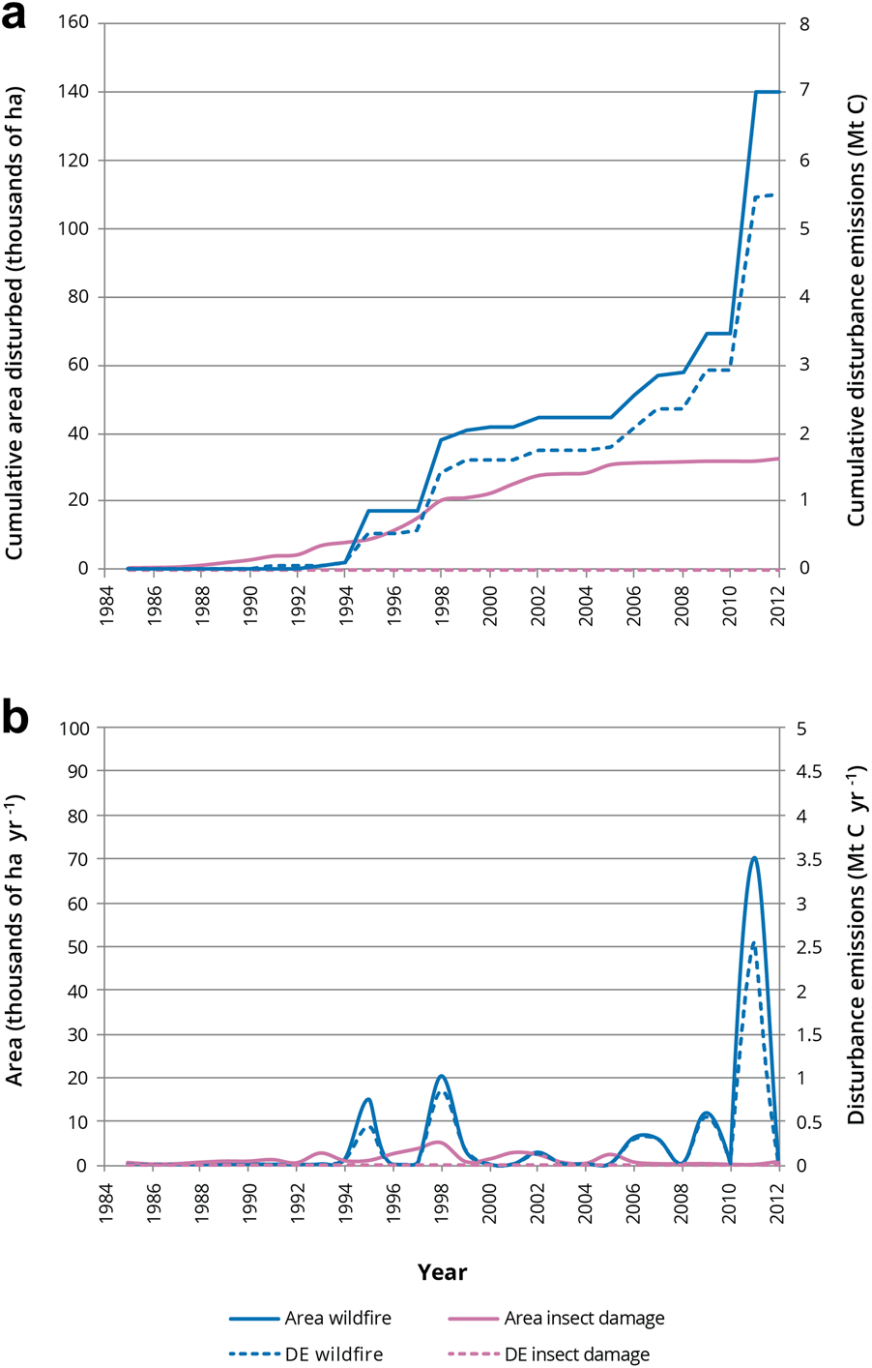
Cumulative disturbance emissions (DE) and area disturbed (**a**), and annual disturbance emissions and area disturbed (**b**) in the pilot study area (1985–2012) from natural disturbance types (wildfire, insect disturbances)

**Fig. 7:**
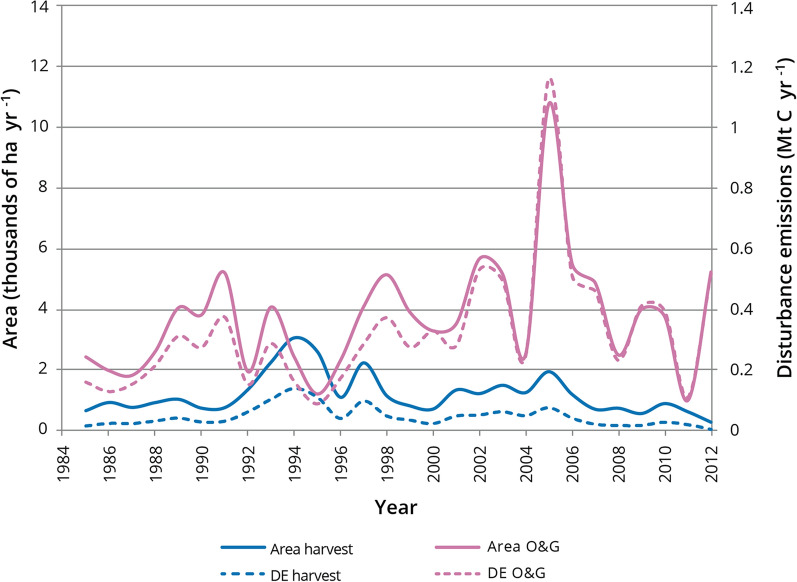
Annual disturbance emissions (DE) and area disturbed in the pilot study area (1985–2012) from forest (harvest) and oil and gas (O&G) sectors

Approximately 25% (312,864.4 ha) of the pilot study area was disturbed over the simulation period of 28 years. Forty-five percent of the disturbed area was affected by anthropogenic disturbances and 55% by natural disturbances (Fig. 5a). We examined two different natural disturbance types; wildfire and insect disturbances (e.g., defoliation, bark beetle). The percentage of the naturally disturbed area affected by wildfire (81%) was much larger than that affected by insect disturbances (19%) (Fig. 6a). The cumulative annual area disturbed by insects steadily increased from 1985 to 2005 and then leveled off to 2012, whereas fire disturbance events were episodic and appear as spikes in large fire years (Fig. 6). Anthropogenic disturbances that we examined included harvesting plus seven different oil and gas development-related disturbance types. Slightly more area (141,171 ha) was affected by anthropogenic disturbances than by wildfire (139,365 ha). Harvesting disturbed a much smaller percentage of the anthropogenically disturbed area (22.2%) than oil and gas related disturbances (73.8%). With the exception of two years (1994 and 1995) in the simulation period, the area disturbed by oil and gas activities was always greater than that disturbed by harvesting (Fig. 7). Over time the annual changes in anthropogenically disturbed areas were characterized by large peaks and troughs (Fig. 7), likely in response to changes in economic conditions. Within the oil and gas disturbance types, surface mines disturbed the largest area (43%), followed by unspecified in-situ (23%), seismic lines (14%), permanent well pads and roads (11%), and aboveground pipelines (4%).

### Ecosystem carbon stocks and fluxes

**Fig. 8:**
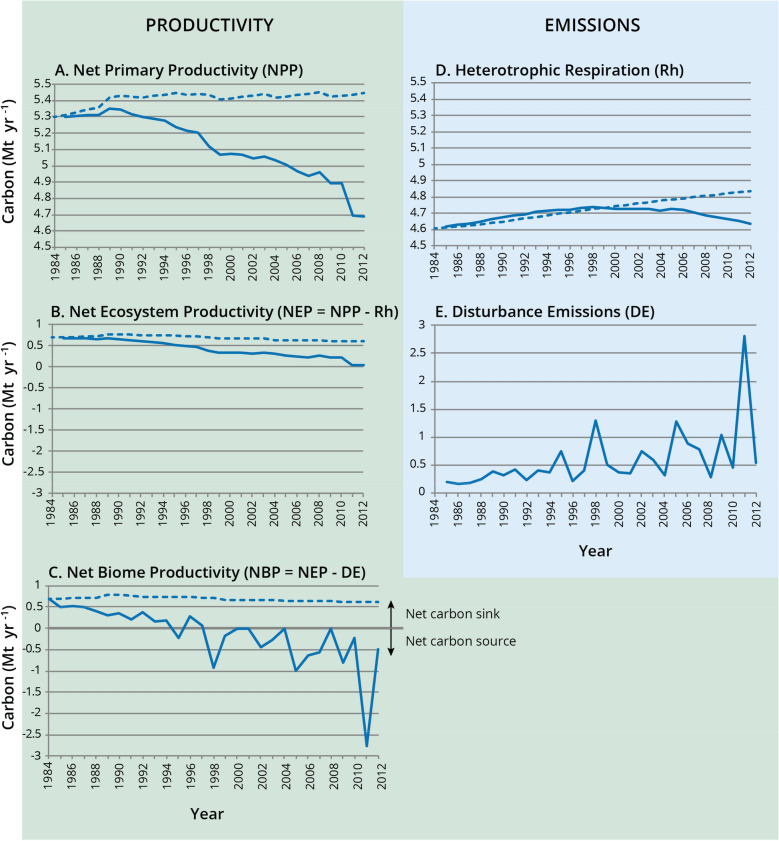
Net productivity (net primary productivity, NPP; net ecosystem productivity, NEP; net biome productivity, NBP) and emission (heterotrophic respiration, Rh; disturbance emissions, DE) ecosystem indicators for the pilot study area (1985–2012) with no disturbances (theoretical, dashed lines) and all simulated disturbances (solid line)

In the pilot study area, total modelled ecosystem C stocks decreased by about 4.7 Mt over the simulation period from about 332.2 Mt to about 327.5 Mt, an average loss rate of 0.128 t C ha^−1^ yr^−1^. Above and belowground biomass decreased by 2.9 Mt C. Deadwood and organic soil pools decreased by 3.3 Mt C while mineral soil C increased by 1.5 Mt, or 1.2% over 28 years. Reductions in C stocks over time are related to disturbance types that have large or consistent effects on ecosystem emissions flux indicators. Ecosystem flux indicator outputs from the GCBM include net primary productivity (NPP), heterotrophic respiration (Rh), net ecosystem productivity (NEP = NPP − Rh), disturbance emissions (DE), and net biome productivity (NBP = NEP − DE). In the absence of disturbances (theoretical, as natural disturbances are integral to boreal ecosystems in Canada) the pilot study area would have remained a relatively constant C sink (positive values for NBP) (Fig. 8). When all disturbances were included, NPP and NEP declined over time as the area supporting forest decreased. Consequently, the study area gradually changed from a sink to a source (positive to negative NBP) with episodic peaks of DEs and negative NBP that mainly correspond to large wildfire disturbance events (Fig. 8c). The overall downward trend in NBP is related to a decline in NPP (Fig. 8a) and therefore NEP (Fig. 8b) that is exacerbated by a gradual increase in DEs (Fig. 8e).

Annual DEs from anthropogenic and natural disturbances follow the pattern of annual areas disturbed by each type (Fig. 5b). Although the annual area affected by anthropogenic disturbances is lower than that for natural disturbances, annual emissions from anthropogenic disturbances are higher than for natural disturbances, except in peak years for natural disturbances that are associated with large wildfires (Fig. 5b). The net result is that the cumulative area affected by disturbances is larger for natural than for anthropogenic disturbances but the cumulative DEs are larger for anthropogenic than for natural disturbances (Fig. 5a). In the Ft. McMurray region the frequency of large fires has increased over recent decades (e.g., the large DE peak in 2011 is due to the Richardson fire (700,000 ha [58]) and if the Ft. McMurray fire of 2016 (590,000 ha) [59] had been included in this study the endpoint for cumulative emissions from natural disturbances may have been higher depending on how much of the pilot study area was affected by the Ft. McMurray wildfire (Fig. 5a).

We assessed the two most important natural disturbance types in the study area, insect damage and wildfires. At the end of 2012, the cumulative area disturbed by insect damage was one quarter of the area with wildfire, but the cumulative direct DEs from insect damage were far less than from wildfire (Fig. 6a). This is, in part, because wildfires cause large direct emissions in the year of the fire, while the impacts of insect disturbances on NPP and Rh occur in the years after the disturbance, and are not easily distinguished from annual processes in the model.

**Fig. 9:**
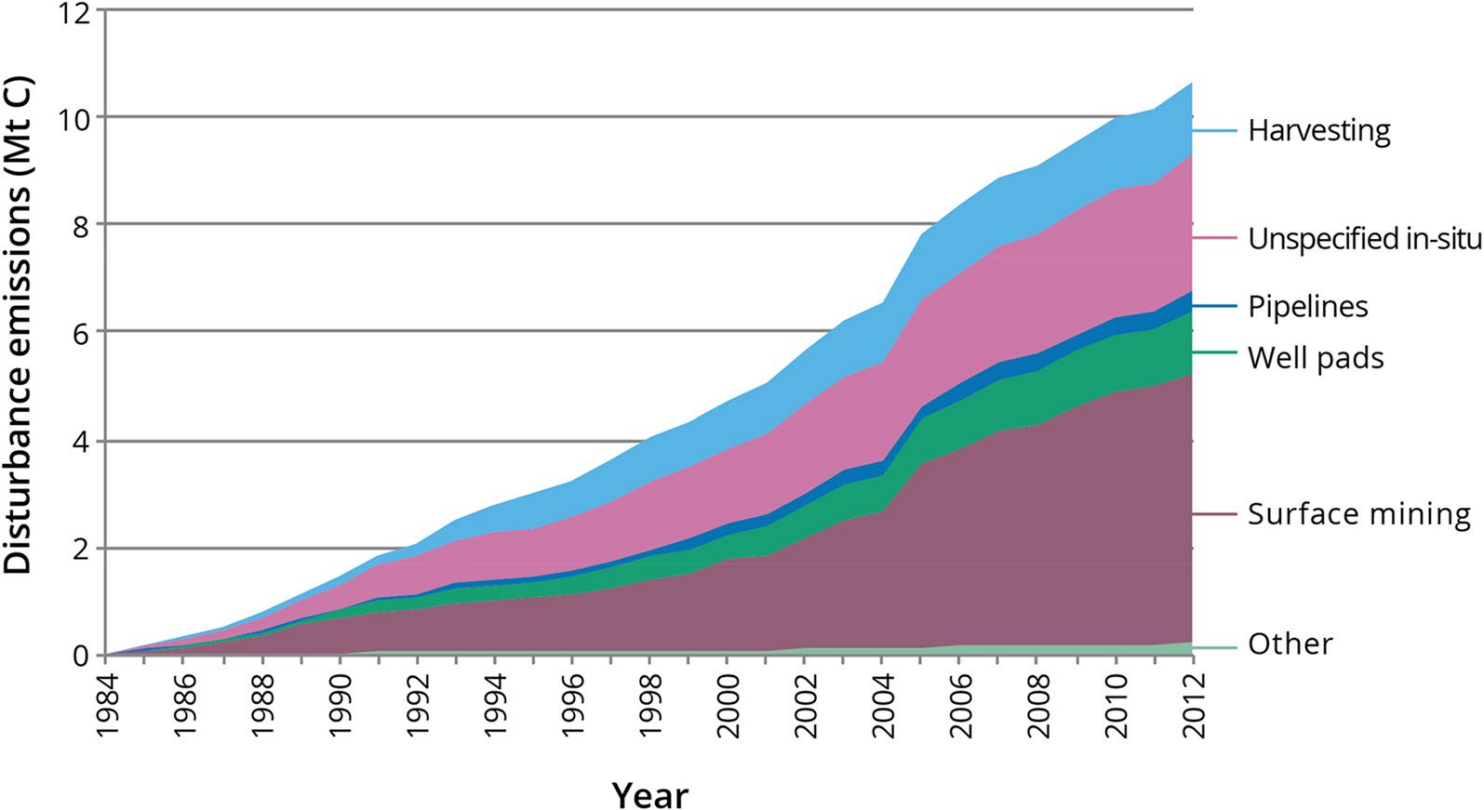
Cumulative disturbance emissions in the pilot study area (1985–2012) from forest harvesting and different types of oil and gas development disturbances. “Other” includes seismic lines, industrial sites, and municipal lands

The two major contributors to anthropogenic disturbances were the forest (harvesting) and energy (oil and gas development) sectors. In each sector, the pattern of annual emissions tracked the pattern for area disturbed (Fig. 7). In most years the annual area disturbed and DEs from forest harvesting were lower than for all oil and gas activities combined (Fig. 7), the exception being 1995 where DEs from harvesting were slightly higher than DEs from oil and gas activities. Cumulative DEs from the energy sector disturbance types were highest for surface mining (5 Mt C), followed by unspecified in-situ (2.6 Mt C), well pads (1.2 Mt C) and then pipelines and other including seismic lines (0.6 Mt C) (Fig. 9). Cumulative DEs from forest harvesting (1.3 Mt C) were slightly higher than for well pads alone. Although these disturbance types had similar cumulative DEs, their contributions to NBP (i.e., whether the area is a sink or source) are different because, in most cases, harvested areas are regrowing and contributing to NPP, while well pads are not as long as the well remains in operation, abandoned and not reclaimed, or if reclamation success is poor. Disturbance emissions from natural disturbances (mainly wildfire) were greater than any individual oil and gas disturbance (Fig. 9) but when all cumulative oil and gas disturbances were summed their DEs were about double the DEs from natural disturbances (Fig. 5a).

Thus, the relative importance of disturbance types on landscape-level cumulative effects in the pilot study area differ when judged only on the basis of area affected, or when judged on the combined effects of area and effect of disturbance type on C fluxes. When judged by area alone, the greatest cumulative effect on the landscape was from wildfire (139,000 ha), followed by the combined oil and gas sector disturbances (110,000 ha; mainly surface mining and unspecified in-situ) and then insect damage (33,000 ha) and harvesting (31,000 ha). From a C balance perspective the impacts on GHG emissions and removals amongst disturbance types differed. Cumulative DEs were greatest from the oil and gas sector (9.5 Mt C, primarily surface mining and unspecified in-situ), followed by wildfire (5.5 Mt C) and then forest harvesting (1.3 Mt C). Disturbance emissions from insect damage were relatively small, as were DEs from seismic lines where the area disturbed was estimated at 15,000 ha.

## Discussion

Assessing cumulative effects of proposed projects on C stocks and fluxes is a requirement of EIAs in Canada. However, for projects proposed in forested areas, and in particular the boreal forest of Canada, tools have not been developed specifically for this purpose so current assessments in EIAs take relatively simple approaches. Here, we demonstrate how an existing tool, the GCBM (spatially-explicit version of the CBM-CFS3), developed by the Canadian Forest Service for national and international forest C reporting, can be used for a more rigorous assessment of the cumulative effects of anthropogenic and natural disturbances on an upland forest C balance to satisfy EIA requirements. In the pilot study area in the OSR of Alberta, we integrated spatially-explicit annual time series of LandSat disturbance data with forest inventory and ecosystem C dynamics modelling by applying the GCBM to approximately 1.3 million ha of upland forest on a 30 m × 30 m (approximate) grid. However, the GCBM can be applied to smaller areas of interest to project proponents, or larger areas for regional analyses that would be of interest to managers or policy makers, using a grid appropriate to the goals of the user [37–39]. Using a spatially-explicit model allows us to generate maps of results that can be verified by independent third parties, using field visits or other remote sensing data.

Our results show that the cumulative effect of anthropogenic and natural disturbances on the forest C balance (1985–2012) was to turn the upland forest in the pilot study area from a net C sink to a net C source. Because of ongoing oil and gas activities and large fire emissions that would come from any part of the pilot study area that was affected by the Ft. McMurray wildfire in 2016 there is no reason to believe that the study area will have reverted to a net C sink by 2020. We concluded that 25% of the pilot study area was disturbed over the 28 year period, or about 0.85% per year. The largest contributors to cumulative DEs were oil and gas disturbances (mainly surface mining and unspecified in-situ disturbances), followed by wildfire and then forest harvesting. Increasing DEs and reductions in forest area, combined with declining NPP over the 28 years of the pilot study, changed the area from a sink to a source. Most disturbances, except those caused by insect damage, contributed in varying degrees to the slow rate of decline in NPP primarily by taking land out of forest production for extended periods of time (e.g., well pads, temporary roads, surface mines), or creating conditions where forests have difficulty re-establishing so that there is a significant lag in re-establishing NPP, or returning forest stands to early stages of growth when NPP is low (e.g., recent wildfires or harvesting). The oil and gas sector was the major contributor to deforestation (i.e., land-use change). All oil and gas disturbances would meet the definition of deforestation except seismic lines and unspecified in-situ disturbances because of their narrow width. These fine scale disturbances, collectively over space and time in the pilot study area, accounted for 13% of the disturbed area, and 16% of the disturbance emissions.

This was a retrospective study with complete historical data that allowed us to look back over a 28-year period of time where the pilot study area was subjected to intense disturbances, to demonstrate the type of analysis, useful for EIAs, which can be achieved with the spatially-explicit GCBM. This type of retrospective analysis could be useful to policy makers interested in regional assessments to understand what activities within sectors have contributed to tipping the regional C balance from a sink to a source. However, the GCBM (or CBM-CFS3) can also be use in scenario analyses to assess the net impact of future anthropogenic activities on forest emissions and removals (e.g., change relative to a baseline) and the value of different approaches to management or policy to mitigate negative outcomes of cumulative effects, similar to mitigation strategy analyses that have been done for the forest sector [34, 60, 61]. Using this approach, project proponents could test alternate management strategies to demonstrate how selected management practices could contribute to reducing future GHG emissions. For example, in this analysis C sent to HWPs was treated as an immediate emission, which is one option that is consistent with international accounting rules. However, depending on end use, C can be stored in HWPs for decades or centuries with a result that managing forest product streams to favour long-lived products is one strategy that could contribute to reducing future GHG emissions [34, 62]. This information could inform development of project proposals to reduce or offset potential project impacts on forest emissions and C storage.

We focussed on an area with a high intensity and variety of disturbance types to illustrate the value of using GCBM as a tool for cumulative effects analysis for EIAs. We do not recommend extrapolating the results from this pilot study area to the entire OSR because the dominant types, patterns and intensities of disturbances are highly variable throughout the boreal region of northern Alberta and outcomes will differ depending on the landscape being analyzed. For example, in parts of the OSR that are less affected by oil and gas activities, wildfire and harvesting may be the major contributors to DEs, while in parts of the OSR dominated by in-situ resource extraction rather than surface mining, oil and gas exploration activities may play a dominant role in contributing to DEs. Representation of disturbances at the sub-pixel level, enabled examination of the impacts of multiple linear disturbances on the forest C balance at the landscape scale. This study only included upland forest types, and did not include the contributions of wetlands (peatlands) to net GHG emissions, either in their natural state or once disturbed. Wetlands occupy almost half of the landbase in the entire OSR so their contributions to the regional C balance is expected to be significant [24, 63, 64] because of the large area they occupy, their large C stocks, and how they are affected by climate change, and natural and anthropogenic disturbances. For example, a study by Strack et al. [65], reported increased methane emissions from petroleum exploration disturbances on peatlands in Alberta, Canada, due to local soil compaction and wetter conditions. Also, in our study we only assessed biogenic emissions—i.e., those related to the upland forests. Emissions from fossil sources associated with industrial development, fugitive emissions and from resource extraction are beyond the scope of this study but are significant and can easily exceed biogenic emissions [66].

We have shown that the GCBM (or CBM-CFS3) can be used for a C assessment suitable to meet the requirements of EIAs in Canada and this approach could also be used in monitoring of cumulative effects on forest C emissions and removals. Regardless of the application, the system is only as good as the data used for input and science used to inform the model. For forest C assessments in the OSR, improvements would come from better identification of sub-pixel sized disturbances from oil and gas activities, better data on the year of the disturbance event, enhanced spatial forest inventories, spatial records for timing and type of reclamation and restoration activities and their success rates of re-establishing the ecosystem C sink function. Increased field research on the GHG and C impacts of various disturbances, such as soil disturbances, would enhance parameterization and validation of the model. Improved detection of the rate of post disturbance recovery, and research to improve our understanding of forest recovery in response to different management practices would be extremely useful to improving accuracy in predicted net ecosystem emissions. For example, contributions of seismic lines may be greater than shown in our pilot study, because we assume that forest stands recover after disturbance, whereas it has been documented, especially for legacy seismic lines, that regrowth of forests may be significantly delayed, often due to repeated use of these lines by off-road vehicles [57]. The impact of significant delays or failure of forests to re-establish would be to reduce NPP and likely increase net emissions of GHGs to the atmosphere. Other potential negative impacts, such as reductions in NPP near surface mining have been observed but were not quantified here [67].

## Conclusions

Currently project proponents submitting EIAs to support oil and gas development in Canada are lacking the tools required to conduct a thorough analysis of the cumulative effects of a project on forest C. We have demonstrated that the GCBM can be applied to achieve a regional assessment of cumulative effects of natural and anthropogenic disturbances at the fine scale required to include disturbances from oil and gas exploration and extraction. We found that our pilot study area turned from a net C sink to a net C source over the 28 year assessment period. This change occurred as a result of both increases in DEs and decreases in NPP. Increases in DEs occurred through increases in the frequency of wildfires and the cumulative effect of oil and gas activities causing emissions through combustion or disturbance of biomass, dead organic matter or soil. Reductions in NPP resulted from forest land being taken out of production, or returning forests to early stages of stand growth that have low NPP. The system we used can be easily adapted to smaller areas and finer grids to address the goals of users, but the accuracy of analyses at finer scales is dependent on the resolution of input data layers and available C science. This pilot study was applied only to the upland forest component of the landscape. Complete assessments will need to include the contributions of wetlands (peatlands), which can be addressed using the Canadian Model for Peatlands (CaMP) that has been developed for applications within the GCBM framework [68].

## Supplementary Information

**Additional file 1: Appendix S1**. Disturbance matrices developed for common oil and gas exploration and development disturbance types in the pilot study area.

## Data Availability

The datasets analysed during the current study are available from the corresponding author on reasonable request.
